# The Incidence of Deep Vein Thrombosis in Breast Cancer Patients Receiving Outpatient Cancer Therapy in Iran

**Published:** 2019-03

**Authors:** Babak Sharif-Kashani, Ali Ghanbari Motlagh, Ahmad R Mafi, Omid Esnaashari, Mani Ramzi, Ali Taghizadeh, Safa Najafi

**Affiliations:** 1 Tobacco Prevention and Control Research Center, National Research Institute of Tuberculosis and Lung Disease (NRITLD), Shahid Beheshti University of Medical Sciences, Tehran, Iran,; 2 Department of Radiation Oncology, Imam Hossein Hospital, Shahid Beheshti University of Medical Sciences, Tehran, Iran,; 3 Urmia Omid Hospital, Radiation and Oncology Center, Urmia, Iran,; 4 Department of Medical Oncology and Hematology and Bone Marrow Transplantation, Hematology Research Center, Shiraz University of Medical Sciences, Shiraz, Iran,; 5 Department of Radiation Oncology, Imam Reza University Hospital, Mashhad, Iran,; 6 Breast Diseases Department, Breast Cancer Research Center, Motamed Cancer Institute, ACECR, Tehran, Iran.

**Keywords:** Deep vein thrombosis, Breast cancer, Outpatient, Chemotherapy

## Abstract

**Background:**

Venous thromboembolism (VTE) is one of the main causes of mortality in patients with cancer. This study was conducted to assess the incidence of deep vein thrombosis (DVT) in breast cancer patients receiving outpatient cancer therapy.

**Materials and Methods:**

This multi-center prospective cohort study was conducted on patients with breast cancer, initiating an outpatient chemotherapy regimen in five medical centers in Iran. Eligible patients were enrolled in the study consecutively between January 2013 and January 2015. The primary outcome was lower extremity DVT based on duplex/doppler ultrasonography two months after the first course of chemotherapy (visit 2) and after the end of the course (visit 3). All patients were followed-up from the onset of chemotherapy until the first occurrence of lower extremity DVT, death, or the end of the course.

**Results:**

A total of 427 eligible breast cancer patients were recruited in the study, 403 of whom attended at least one follow-up visit. The mean (SD) duration of follow-up was 4 (1.3) months. During the follow-up, only one patient showed DVT on duplex/doppler ultrasonography in visit 2. Therefore, the two-month and overall cumulative incidence risk of DVT was 0.25% (95% CI: 0.00–0.74%). However, the mean D-dimer level showed no significant change (P>0.05).

**Conclusion:**

Our findings showed the low risk of DVT in breast cancer patients receiving outpatient cancer therapy.

## INTRODUCTION

Venous thromboembolism (VTE) is a major health problem among patients with cancer. Evidence shows that VTE can be associated with a two- to six-fold increase in the risk of mortality in these patients (1–4). In cancer patients, comorbidities, advanced age, genetic syndromes (e.g., anti-phospholipid syndrome and anti-thrombin III deficiency), and disease-related factors (e.g., type of malignancy, malignancy stage, and presence of metastasis) can define the risk of VTE (4–6). Furthermore, treatment-related factors can affect the incidence of VTE, including type of surgery and type of systemic therapy (7).

Breast cancer is among the most common malignancies worldwide (8). A number of previous studies have shown that women with this disease may have a three- to four-fold increased risk of VTE, compared to women without cancer in the same age group (9). Given the high incidence of breast cancer among women worldwide (8), even a small increase in the occurrence of VTE in these patients can have a substantial impact on public health and medical resources (9, 10).

To the best of our knowledge, only one recently published study has assessed the incidence of deep venous thrombosis (DVT) in outpatients with breast cancer (11). Therefore, the purpose of the present study was to assess the incidence of lower extremity DVT in breast cancer outpatients receiving outpatient cancer therapy.

## MATERIALS AND METHODS

### Study design and participants

This prospective multi-center cohort study was conducted on patients with breast cancer, who were scheduled to start outpatient systemic treatment in five medical centers of Iran. The study was performed in four hospitals and one private clinic, including Omid Hospital (Urmia), Namazi Hospital (Shiraz), Imam Hossein Hospital (Tehran), Imam Reza Hospital (Mashhad), and Dr. Najafi Private Clinic (Tehran). Breast cancer patients referred to these centers were included if they met the following criteria: 1) written informed consent; and 2) plan for initiation of outpatient cancer therapy within one month. On the other hand, the exclusion criteria were as follows: 1) a history of hospitalization and/or surgery within two weeks before the study; 2) hospitalization for more than three days during the study; 3) a documented acute DVT at baseline (or ongoing prophylaxis for VTE at the time of diagnosis); 4) receiving anticoagulants, hormone replacement therapy (HRT), or oral contraceptive pills (OCP); 5) metastasis with an Eastern Cooperative Oncology Group-Performance Status (ECOG-PS) score ≥3; and 6) undergoing chemoprophylaxis up to one month before the study. Eligible patients were enrolled in the study consecutively between January 2013 and January 2015 and assessed at the onset of the chemotherapy course (baseline, visit 1), two months after the first course of chemotherapy (first follow-up, visit 2), and almost one week after (or before) the end of the course (second follow-up, visit 3).

This study was reviewed and approved by the Ethics Committee of Masih Daneshvari Hospital in Tehran, Iran. In accordance with the ethics committee requirements and Declaration of Helsinki by the World Medical Association (WMA), all patients received the necessary information about the study before they were asked to sign a consent form if they were willing to participate. Patients could withdraw consent at any time throughout the study for any reason. All patients’ data remained anonymous in the study.

### Data collection

In baseline and follow-up visits, all necessary data were collected from the patients’ medical files and records and transferred into a case report form (CRF), which was developed a priori by trained nurses. The recorded data on CRFs were submitted to a central coordinating center. All collected data are listed below:

**In the first visit (baseline):**
- Patients’ characteristics: year of birth, weight, height, marital status, menopausal status, place of residence (city), and anti-platelet drug use
- Presence of comorbidities, such as hypertension, stroke/transient ischemic disease, diabetes mellitus, congestive heart failure, pulmonary disease, hepatic disease, atrial fibrillation/flutter, chronic kidney disease, and others, all identified based on the International Classification of Diseases, Ninth Revision, Clinical Modification (ICD-9-CM)
- Clinical characteristics of breast cancer, including the side (unilateral or bilateral), predominant histological type, TNM stage and grade of primary tumor, and metastasis
- Results of biomarker tests, including HER2/neu/ErbB2 and estrogen and progesterone receptors
- Recognized risk factors for DVT, such as pregnancy, puerperium, central venous catheters, family history of DVT, previous DVT, inherited/acquired hemophilia, inflammatory bowel disease, nephrotic syndrome, trauma (major or lower limbs), current smoking, and body mass index (BMI) >30 kg/m ^2^ .
- Laboratory test results, including complete blood count (platelet, hemoglobin, white blood cell, and polymorphonuclear neutrophil count) and D-dimer level (µg/L FEU)

**In visit 2 and 3 (follow-up visits):**
- Main details of the chemotherapy plan, including drugs, number of cycles, and date of administration
- Main details of breast cancer surgery, including the date, type, and procedure of surgery
- Main details of radiotherapy, including the date, number of fields and fractions, and total dose
- Main details of hormone therapy, such as the starting date and used drugs
- Main details of hospitalization, such as the date, duration, and main cause of admission
- Results of lower extremity Doppler ultrasonography (DVT as the outcome)
- Main details of follow-ups, including the date of the first and last visits, treatment received for DVT, and the used drugs, doses, and duration
- Cancer progression and survival data.

### Data quality control

The quality of the recorded data was controlled by comparing the collected data with the medical records of patients of all included centers after each visit. In addition, the plausibility and completeness of the key transferred data were checked randomly and regularly by data managers at the coordinating center. The centers were contacted in the event of any discrepancy or missing value to ask for modification or verification of the data. The study sites were visited regularly by clinical examiners to check for informed consents and key data.

### Variable measurements

BMI was calculated based on the measured height and weight (kg/m ^2^) in the baseline visit and was classified into five categories: ≤18.5 kg/m ^2^ (underweight), 18.6–24.9 kg/m ^2^ (normal), 25.0–29.9 kg/m ^2^ (overweight), 30.0–34.9 kg/m ^2^ (obese), and ≥35.0 kg/m ^2^ (severely obese). The TNM staging was converted into summary stages, including localized, regional, and distant or unknown according to the algorithm proposed by Ording et al. (12). Moreover, endocrine treatment types were classified into two categories: selective estrogen receptor modulator (SERM) and newer-generation aromatase inhibitors (AIs). The number of comorbidities was also calculated for each patient, based on the medical records.

In addition, in order to calculate the baseline VTE risk score (VTE-RS) based on the method developed by Khorana et al. (13), the following continuous variables were converted into binary variables for each patient: BMI (≥35 vs. <35 kg/m ^2^), platelet count (≥350 vs. <350×10 ^9^ /L), hemoglobin (<10 vs. ≥10 g/dL), and leukocyte count (<11 vs. ≥11×10 ^9^ /L). It should be noted that a Khorana risk score of zero indicates the low risk of breast cancer (as applied in our cohort).

### Outcome measurements

The main outcome was the incidence of DVT during chemotherapy in breast cancer outpatients. In each follow-up visit, all patients were screened for DVT by performing lower extremity Doppler ultrasonography and assessing D-dimer regardless of whether the patient was symptomatic or not. DVT was defined using the validated ICD-9-CM diagnostic codes for thrombophlebitis and venous thrombosis of the lower extremity. It was diagnosed based on the clinical symptoms or physical signs and confirmed using the duplex/doppler ultrasonography criteria for DVT by the treating physician.

### Statistical methods and data analysis

The baseline characteristics of the patients were summarized and presented with descriptive statistics, and inter-cohort comparisons were performed. Categorical variables were reported as percentage (absolute frequency) and compared using two-sided Chi-square tests. Continuous variables were reported as mean (standard deviation) and compared using student’s t-tests or repeated measurement tests. P-value less than 0.05 was considered statistically significant. The cumulative incidence risk and 95% confidence intervals were also calculated. All statistical analyses were performed using SAS version 19.0 (SAS Institute Inc., Cary, NC).

## RESULTS

### Descriptive statistics

A total of 427 eligible breast cancer patients were recruited in the study from five centers during 2013–2015. Overall, 403 patients completed the first and/or the second follow-up, 375 of whom attended both follow-ups. The mean (SD) duration of follow-up was four months (SD: 1.3; min: 2; and max: 8 months). The characteristics of patients, tumors, and administered treatments are summarized in Tables 1–3 with respect to the follow-up status and in general.

**Table 1.** Baseline characteristics of the participants in this study, overall and by follow up status

**Characteristic**		**Overall**	**Follow up status**		

			**Completed**	**Non-Completed**	P
		**N: 427**	**N: 375**	**N: 52**	
		**Percent(n) or mean (SD)**	**Percent(n) or mean (S D)**	**Percent(n) or Mea n(SD)**	
Age at diagnosis		48.9 (11.5)	48.7 (11.5)	50.1 (11.4)	0.44
BMI (kg/m^2^) [Missing = 2]	< 18.5	1.2 % (5)	1.3 % (5)	0.0 % (0)	
	18.5 – 25	22.6 % (96)	22.5 % (84)	23.1 % (12)	
	25–30	39.8 % (169)	38.3 % (143)	50.0 % (50)	0.26
	30 – 35	24.5 % (104)	26..0 % (97)	13.5 % (7)	
	≥ 35	12.0 % (51)	11.8 % (44)	13.5 % (7)	
Menopausal status [Missing =4]	Non menopausal	48.5 % (205)	48.8 % (181)	46.2 % (24)	0.77
	Menopausal	51.5 % (218)	51.2 % (190)	53.8 % (28)	
	0	80.9% (343)	82.5 % (307)	69.2 % (36)	
Number of Chronic comorbidity conditions ^*^ [Missing =3]	1	12.0 % (51)	11.6 % (43)	15.4 % (8)	0.06
	2	5.9 % (25)	4.8% (18)	13.5 % (7)	
	3	1.2 % (5)	1.1 % (4)	1.9 % (1)	
Medical history [Missing =1]	Previous VTE	0.7 %(3)	0.8 % (3)	0.0 % (0)	0.52
	Pregnancy or Postpartum	0. 9 % (4)	1.1 % (4)	0.0 % (0)	0.45
	Thrombophilia	1.2 %(5)	1.3 % (5)	0.0 % (0)	0.40
Hemoglobin ^†^ < 10 g/ dl [Missing =25]		5.2 % (21)	4.7 % (17)	9.1 % (4)	0.22
WBCs ^†^ > 11 × 1000/ mm ^3^ [Missing =15]		3.6 % (15)	3.6 % (13)	4.4 % (2)	0.76
Platelets^†^ ≥ 350 × 1000/ mm ^3^ [Missing =16]		27.0 % (111)	27.3 % (100)	24.4 % (11)	0.68
VTE Risk at baseline [Missing =27]	Low risk (Khorana score =0)	58.27 % (233)	58.1 % (207)	59.1 % (26)	
	Intermediate risk (Khorana score= 1 or 2)	41.5 % (166)	41.9 % (149)	38.6 % (17)	0.04
	High risk (Khorana score ≥ 3)	0.3 % (1)	0.0 % (0)	2.3 % (1)	

SD: Standard Deviation, BMI: Body Mass Index VTE: venous thromboembolism

* No body reported history of Central Venous Catheters, Inflammatory Bowel Disease (IBD), Nephrotic syndrome, and Trauma

† Variables used to derive the VTE risk score (together with BMI ≥ 35).

**Table 2.** Clinical characteristics of the participants in this study, overall and by follow up status

**Variables**		**Overall**	**Follow up status**	

			**Completed**	**Non - completed**
		**N: 427**	**N: 375**	**N: 52**
		**Percent (n) or mean (SD)**	**Percent (n) or mean**	**Percent (n) or mean (SD)**
Stage of cancer [Missing =1]	Localized	28.2 % (120)	28.6 % (107)	25.0 % (13)
	Regional	41.2 % (176)	41.7 % (156)	38.5 % (20)
	Distant	4.5 % (19)	4.5 % (17)	3.8 % (2)
	Unknown	26.1 % (111)	25.2 % (94)	32.7 % (17)
	Ductal	91.6 % (370)	91.6 % (328)	91.3 % (42)
	Lobular	4.0 % (16)	4.2 % (15)	2.2 % (1)
Histological subtype of carcinoma [Missing =23]	Medullary	2.2 % (9)	2.0 % (7)	4.3 % (2)
	Mucinous	0.2 % (1)	1.1 % (4)	0.0 % (0)
	Mixed	1.0 % (4)	0.8 % (3)	2.2 % (1)
	Other type	1.0 (4)	0.3 % (1)	0.0 % (0)
	Could not be assessed	3.1 % (8)	2.6 % (6)	8.3 % (2)
Grade [Missing =172]	Well differentiated	27.9 % (71)	28.6 % (66)	20.8 % (5)
	Moderately differentiated	51.4 % (131)	51.1 % (118)	54.2 % (13)
	Poorly/Un differentiated	17.6 % (45)	17.7 % (41)	16.7 % (4)
Estrogen receptor [Missing =20]	Negative	24.8 % (101)	24.9 % (90)	23.9 % (11)
	Positive	75.2 % (306)	75.1 % (271)	76.1 % (35)
Progesterone receptor [Missing =23]	Negative	31.7 % (128)	31.9 % (115)	30.2 % (13)
	Positive	68.3 % (276)	68.1 % (246)	69.8 % (30)

SD: Standard deviation; all p value > 0.05

**Table 3.** Administered therapies, overall and by follow up status

**Variables**		**Overall**	**Follow up status**	

		**N: 427**	**Completed**	**Non -completed**
		**Percent(n)**	**N: 375**	**N: 52**
			**Percent (n)**	**Percent (n)**
**Chemotherapy**		All	All	All
Radiotherapy	Yes	68.7 (274)	67.2 % (252)	44.0 % (22)
[Missing = 28]	No	31.3 % (125)	25.8 % (97)	56.0 % (28)
**Hormone therapy in N= 220**				
Type	A selective estrogen receptor modulator (SERM)	66.8 % (145)	66.5 % (131)	70.0 % (14)
[Missing = 3]	Aromatase inhibitors (AIs)	33.2 (72)	33.5 % (66)	30.0 % (6)
**Surgery in N = 416**				
Type	BCS ^*^	31.7 % (132)	31.7 % (116)	32.0 % (16)
[Missing = 5]	MRM^†^	68.3 % (284)	68.3 % (250)	68.0 % (34)
	Axillary Dissection (AxD)	81.5 % (322)	8.6 % (279)	87.8 %(43)
**Procedure at surgery**				
[Missing = 27]	Sentinel lymph node (SLN) biopsy	13.7 % (54)	14.2 % (49)	10.2 % (5)
	AxD & SLN biopsy	4.8 % (19)	5.2(18)	2.0(1)

* Breast conserving surgery

† Modified radical mastectomy All p value > 0.05

The mean age (SD) of the patients, who attended at least one of the follow-up visits (n=403), was 48.7 years (SD: 11.3; min: 8 years; and max: 80 years) at the time of breast cancer diagnosis; more than half of these patients were post-menopausal (50.9%). Three patients reported a history of VTE, four were pregnant, three had thrombophilia, and one was a cigarette smoker. In addition, 70 (17.5%) patients had at least one major comorbidity, including diabetes (n=34), hypertension (n=47), ischemic heart disease (n=6), cardiac failure (n=1), chronic kidney disease (n=1), and other comorbidities (n=9); some patients had more than one comorbidity.

The Khorana VTE-RS was 0 (low risk) in 55.1% of the patients, 1 or 2 (intermediate risk) in 39.5% of the patients, and ≥3 (high risk) in 0.0% of the patients. However, it could not be calculated in 22 (6.3 %) patients due to lack of data. The only patient categorized as high risk at baseline did not complete the follow-ups and was classified in the incomplete follow-up group. Based on the findings, 380 (94.3%) patients had unilateral breast cancer, and 23 (5.7%) were diagnosed with metastatic breast cancer. Most of the tumors were ductal carcinoma (n=350; 91.6%), and 42 (17.4%) patients were diagnosed with poorly or undifferentiated carcinoma. No data on tumor type were provided in 161 patients.

All patients received chemotherapy. Overall, 215 (51.5%) patients with ER-positive and/or PgR-positive tumors were prescribed endocrine therapy, mostly a selective estrogen receptor modulator (SERM) (n=143; 66.5%). A total of 394 (99%) patients underwent surgical resection, mainly modified radical mastectomy (MRM) (n=265; 67.3%). Comparison of the baseline characteristics between the patients who completed both follow-ups (n=375) and those who did (n=52) revealed that there was no significant difference between the two groups in terms of the baseline characteristics, including age, BMI, number of chronic comorbidities, history of VTE, and characteristics of tumor and therapies (Table 1; P>0.05 for all).

### Cumulative incidence risk of DVT

At baseline, there was no evidence of DVT in 427 breast cancer patients. Among 400 patients who completed the first follow-up (two-month follow-up), only one patient presented with symptoms suggestive of DVT, including pain and edema in the right leg. Diagnosis of distal lower-extremity DVT was confirmed in this patient, using duplex/doppler ultrasonography. In other patients, duplex/doppler ultrasonography of the lower extremities showed no evidence of DVT. Of 378 patients who completed the second follow-up (±1 week after the end of the course), three patients had symptoms suggestive of DVT. However, apart from the patient who was diagnosed with DVT in the first follow-up visit, doppler ultrasonography of the lower extremities did not confirm the diagnosis of DVT in other patients. Therefore, the cumulative incidence risk of DVT during the follow-up (two-month and total) was 0.25% (95% CI: 0.00–0.74%).

### D-dimer measurements during follow-ups

In patients who attended at least one follow-up visit, the mean (SD) D-dimer level (ng/mL) at baseline, in the first follow-up, and in the second follow-up was 5.44 (0.89), 5.34 (0.80), and 5.36 (0.87), respectively (P=0.97).

### Details of the case diagnosed with DVT

The patient was a 62-year-old post-menopausal woman, who was diagnosed with unilateral breast cancer with metastasis to the bone at the time of diagnosis. She had undergone MRM with axillary dissection, which showed a 2.5-cm ductal carcinoma, with negative estrogen, progesterone, and Her2/neu receptors. She had no comorbidities or history of DVT, thrombophilia, or cigarette smoking. Her baseline VTE risk was low, based on the Khorana cancer-associated VTE-RS (baseline platelet count: 382×10 ^9^ /L; hemoglobin: 11.30 g/L; leukocyte count: 7×10 ^9^ /L; BMI: 25.2 kg/m ^2^ ; and d-dimer level: 0.20 mg/L). The lower-extremity doppler examinations showed no DVT at baseline.

The patient received sequential regimens of chemotherapy after surgery. She presented with pain and swelling in the right leg, and her D-dimer level increased to 0.50 mg/L two months after the anthracycline-based regimen. Distal lower-extremity DVT was confirmed by doppler examination in the first follow-up visit. She was initially treated with low-molecular-weight heparin and subsequently with an oral vitamin K antagonist. During the second follow-up (four months after the first follow-up), she experienced pain and swelling in the same leg again, and the duplex/doppler sonogram was positive for a proximal lower-extremity DVT.

## DISCUSSION

In this prospective cohort study, the incidence of clinical DVT in breast cancer patients receiving outpatient chemotherapy was approximately 0.25%, which was lower than expected. In line with our findings, some other studies have also reported the low incidence risk of VTE (0.18–0.28%) even after one or two months of surgery in patients with breast cancer (2, 14). Due to the low incidence rate of the outcome (DVT), we were unable to assess the effect of risk factors.

Generally, previous studies have reported different risks of VTE events in patients with breast cancer (Table 4). This is probably due to differences in the baseline and clinical characteristics of the patients, treatment-related factors, prophylactic interventions, diagnostic approaches, and/or duration of follow-up (15). Therefore, the risk of VTE events has not been precisely determined in breast cancer patients.

**Table 4.** Previous study on the incidence of venous thromboembolism events in breast cancer patients

**First Author (year)**	**Country**	**Breast Cancer Patients (BC)**	**Sample size**	**Mean (SD) or median age**	**Follow up**		**Outcome**		

					**Start**	Duration^*^	**Deep vein thrombosis (DVT)**	**pulmonary embolism (PE)**	**Venous Thromboembolism (VTE)**
Chew(14) (2006)	USA	All	108,255	62.0 (16)	Diagnosis	2 Y	-	-	1.2%
		Asian American	7,490				-	-	0.3%
Andtbacka(2) (2006)	USA	underwent surgery	3898	54.4 (median)	Surgery	2 M	0.10% (4)	0.10% (4)	0.18% (7)
De Martino(16) (2012)	Lebanon	underwent surgery	7,754	-	Surgery	1 M	0.19%	0.12 %	0.28 %
Chen(17) (2014)	Taiwan	Tamoxifen user	17,874	51.4 (11.5)	Diagnosis	1 /2 /7 Y	0.8/1.1/2.51%	0.07/ 0.1 / 0.32%	-
		Non - Tamoxifen user	10,155	53.7 (11.3)		1 /2 /7 Y	0.77 / 1.12 /2.58%	0.06 / 0.13/ 0.32%	-
Walker(18) (2016)	UK	All	13,202	62 median	Diagnosis	5.3 Y c	2.4%	2.1%	4.6%
Gerotziafas(11) (2017)	International	Outpatients	629	55.0 (12)	Enrollment	1 y	6.5 %	0.95 %	9.2 %
Brand (19) (2017)	Sweden	Early BC	8,338	57.1 (10.3)	Diagnosis	1 / 2 / 5 Y c	-	-	2.0 /2.5/ 4.0%
						5.3 / 7.2 Y c	2.8 / 3.1%	1. 7 / 2.1 %	4.4 / 5.1%
Momeni (20) (2018)	USA	underwent surgery	52,547	59.8 (14.1)	Surgery	3 M	-	-	0.8%
Cui(21) (2018)	China	underwent surgery	234	50.0 (9.2)	Surgery	1 Y	6.4 %	-	-
		White W >= 65 Y	251,945	75.5 (7.4)		61.6 (41.0)d M	4.9%	2.8%	6.4%
Faiz (22) 2018	2018	Black W >= 65 Y	24,083	74.7 (7.4)	Diagnosis	54.1 (39.4) d M	7.9%	4.3%	10.1%

Sd standard deviation;

* C: median, d: mean (sd), M: month, Y: year

All three elements of the Virchow’s triad, including venous stasis, hypercoagulability, and vessel wall injury, have been proposed to be involved in the pathogenesis of cancer-associated VTE (23). Malignancy itself induces a state of hypercoagulability to some extent by the release of procoagulant factors from malignant tissues, induction of inflammatory responses, and also inhibition of fibrinolytic activity (24). In addition, cancer treatments (e.g., surgery, chemotherapy, hormonal therapy, and radiotherapy) increase immobility due to advanced disease or debilitating side effects of treatment. Moreover, use of venous catheters for chemotherapy can promote the hypercoagulable state in cancer patients (25–27).

There are several potential explanations for the low VTE rate in our study. First, patients with breast cancer experience a relatively low incidence of VTE events, compared to patients with lymphoma, brain tumors, and pancreatic, stomach, or lung cancer (7, 28). Second, previous studies have shown that a higher stage of disease and older age are both among strong predictors of VTE development (29). Compared to previous studies reporting a higher risk of VTE, our patients were younger, and fewer cases were diagnosed with metastatic cancer.

Furthermore, a short follow-up may lead to an underestimation of the exact incidence. However, evidence suggests that the risk of VTE is the highest in the first three months after diagnosis (15). In addition, Walker et al. (18) found that in breast cancer patients, the effect of chemotherapy on the incidence of VTE is probably limited to the period of active treatment. Most of our patients were followed-up to the end of the last chemotherapy course. Other possible explanations for the low incidence of DVT in our study may be exclusion of patients receiving anticoagulants before enrollment in the study, as well as patients hospitalized for more than three days during the study; these factors could have influenced our results.

Additionally, DVT was primarily screened based on duplex/doppler sonography of the lower extremities in our study. There might be some patients with false negative duplex/doppler sonography results who were not included in the analysis of DVT incidence. In this study, no increment was found in the mean D-dimer level in the follow-up visits, compared to the first visit. Nevertheless, the patient who developed DVT in our cohort had a slightly elevated level of D-dimer compared with the baseline. In this regard, a longitudinal study on patients with colorectal, lung, pancreatic, or brain cancer reported that patients who developed VTE showed a significantly elevated level of D-dimer during a 250-day follow-up, compared to those who did not (30).

In general, development of VTE in cancer patients is known to be associated with an increase in morbidity, mortality, and medical costs (15). Although systemic VTE prophylaxis is not routinely recommended in breast cancer patients due to the relatively low incidence of VTE and the increased risk of bleeding in this group (31, 32), high-risk patients would potentially benefit from early detection of the problem (18, 19). However, breast cancer patients are all advised to use mechanical anti-embolism devices along with early ambulation in the postoperative period (29). In addition, the only patient who was categorized as high-risk based on the Khorana VTE-RS at baseline, missed both follow-ups, and this missing data could have affected our results.

## CONCLUSION

Based on our finding, the incidence of clinical DVT in breast cancer patients receiving outpatient chemotherapy is low. Therefore, routine use of thromboprophylaxis in these patients is not recommended.

## References

[1] Deitcher SR. Cancer-related deep venous thrombosis: clinical importance, treatment challenges, and management strategies. Semin Thromb Hemost 2003;29(3):247–58. 12888929 10.1055/s-2003-40963

[2] Andtbacka RH Babiera G Singletary SE Hunt KK Meric-Bernstam F Feig BW Incidence and prevention of venous thromboembolism in patients undergoing breast cancer surgery and treated according to clinical pathways. Ann Surg 2006;243(1):96–101. 16371742 10.1097/01.sla.0000193832.40178.0a PMC1449977

[3] Seddighzadeh A Shetty R Goldhaber SZ. Venous thromboembolism in patients with active cancer. Thromb Haemost 2007;98(3):656–61. 17849056

[4] Khalil J Bensaid B Elkacemi H Afif M Bensaid Y Kebdani T Venous thromboembolism in cancer patients: an underestimated major health problem. World J Surg Oncol 2015;13:204. 26092573 10.1186/s12957-015-0592-8 PMC4486121

[5] Wun T White RH. Epidemiology of cancer-related venous thromboembolism. Best Pract Res Clin Haematol 2009;22(1):9–23. 19285269 10.1016/j.beha.2008.12.001 PMC2702321

[6] Zahir MN Shaikh Q Shabbir-Moosajee M Jabbar AA. Incidence of Venous Thromboembolism in cancer patients treated with Cisplatin based chemotherapy - a cohort study. BMC Cancer 2017;17(1):57. 28093087 10.1186/s12885-016-3032-4 PMC5238519

[7] Horsted F West J Grainge MJ. Risk of venous thromboembolism in patients with cancer: a systematic review and meta-analysis. PLoS Med 2012;9(7):e1001275. 10.1371/journal.pmed.1001275 PMC3409130 22859911

[8] Ferlay J Soerjomataram I Dikshit R Eser S Mathers C Rebelo M Cancer incidence and mortality worldwide: sources, methods and major patterns in GLOBOCAN 2012. Int J Cancer 2015;136(5):E359–86. 25220842 10.1002/ijc.29210

[9] Walker AJ Card TR West J Crooks C Grainge MJ. Incidence of venous thromboembolism in patients with cancer - a cohort study using linked United Kingdom databases. Eur J Cancer 2013;49(6):1404–13. 23146958 10.1016/j.ejca.2012.10.021

[10] Falanga A Zacharski L. Deep vein thrombosis in cancer: the scale of the problem and approaches to management. Ann Oncol 2005;16(5):696–701. 15802275 10.1093/annonc/mdi165

[11] Gerotziafas GT Taher A Abdel-Razeq H AboElnazar E Spyropoulos AC El Shemmari S A Predictive Score for Thrombosis Associated with Breast, Colorectal, Lung, or Ovarian Cancer: The Prospective COMPASS-Cancer-Associated Thrombosis Study. Oncologist 2017;22(10):1222–1231. 28550032 10.1634/theoncologist.2016-0414 PMC5634762

[12] Ording AG Nielsson MS Frøslev T Friis S Garne JP Søgaard M. Completeness of breast cancer staging in the Danish Cancer Registry, 2004–2009. Clin Epidemiol 2012;4 Suppl 2:11–6. 22936852 10.2147/CLEP.S31574 PMC3429150

[13] Khorana AA Kuderer NM Culakova E Lyman GH Francis CW. Development and validation of a predictive model for chemotherapy-associated thrombosis. Blood 2008;111(10):4902–7. 18216292 10.1182/blood-2007-10-116327 PMC2384124

[14] Chew HK Wun T Harvey DJ Zhou H White RH. Incidence of venous thromboembolism and the impact on survival in breast cancer patients. J Clin Oncol 2007;25(1):70–6. 17194906 10.1200/JCO.2006.07.4393

[15] Donnellan E Khorana AA. Cancer and Venous Thromboembolic Disease: A Review. Oncologist 2017;22(2):199–207. 28174293 10.1634/theoncologist.2016-0214 PMC5330704

[16] De Martino RR Goodney PP Spangler EL Wallaert JB Corriere MA Rzucidlo EM Variation in thromboembolic complications among patients undergoing commonly performed cancer operations. J Vasc Surg 2012;55(4):1035–1040.e4. 22409858 10.1016/j.jvs.2011.10.129 PMC3339376

[17] Chen TW Chen HM Lin CH Huang CS Cheng AL Lai MS No increased venous thromboembolism risk in Asian breast cancer patients receiving adjuvant tamoxifen. Breast Cancer Res Treat 2014;148(1):135–42. 25240736 10.1007/s10549-014-3140-2

[18] Walker AJ West J Card TR Crooks C Kirwan CC Grainge MJ. When are breast cancer patients at highest risk of venous thromboembolism? A cohort study using English health care data. Blood 2016;127(7):849–57; quiz 953. 26574606 10.1182/blood-2015-01-625582 PMC4778298

[19] Brand JS Hedayati E Bhoo-Pathy N Bergh J Hall P Humphreys K Time-dependent risk and predictors of venous thromboembolism in breast cancer patients: A population-based cohort study. Cancer 2017;123(3):468–475. 27727456 10.1002/cncr.30364

[20] Momeni A Fox JP. Venous Thromboembolism After Surgical Treatment of Breast Cancer. Ann Plast Surg 2018;80(2):188–192. 29095189 10.1097/SAP.0000000000001249

[21] Cui LN Li N Fu S Zhang X Wang X Wang RT. Combination of preoperative D-dimer and mean platelet volume predicts postoperative deep venous thrombosis in breast cancer patients. Cancer Biomark 2018;21(4):909–913. 29278886 10.3233/CBM-170975 PMC13078319

[22] Faiz AS Guo S Kaveney A Philipp CS. Venous thrombosis and breast cancer in older women: Racial differences in risk factors and mortality. Thromb Res 2018;171:130–135. 30296717 10.1016/j.thromres.2018.10.002

[23] Kyrle PA Eichinger S. Is Virchow’s triad complete? Blood 2009;114(6):1138–9. 19661277 10.1182/blood-2009-05-223511

[24] Lyman GH Khorana AA. Cancer, clots and consensus: new understanding of an old problem. J Clin Oncol 2009;27(29):4821–6. 19752337 10.1200/JCO.2009.22.3032 PMC2764390

[25] Caine GJ Stonelake PS Rea D Lip GY. Coagulopathic complications in breast cancer. Cancer 2003;98(8):1578–86. 14534872 10.1002/cncr.11702

[26] Kirwan CC McDowell G McCollum CN Byrne GJ. Incidence of venous thromboembolism during chemotherapy for breast cancer: impact on cancer outcome. Anticancer Res 2011;31(6):2383–8. 21737669

[27] Haddad TC Greeno EW. Chemotherapy-induced thrombosis. Thromb Res 2006;118(5):555–68. 16388837 10.1016/j.thromres.2005.10.015

[28] Petterson TM Marks RS Ashrani AA Bailey KR Heit JA. Risk of site-specific cancer in incident venous thromboembolism: a population-based study. Thromb Res 2015;135(3):472–8. 25547213 10.1016/j.thromres.2014.12.013 PMC4339484

[29] Kyriazi V. Breast cancer as an acquired thrombophilic state. J Breast Cancer 2012;15(2):148–56. 22807931 10.4048/jbc.2012.15.2.148 PMC3395737

[30] Reitter EM Kaider A Ay C Quehenberger P Marosi C Zielinski C Longitudinal analysis of hemostasis biomarkers in cancer patients during antitumor treatment. J Thromb Haemost 2016;14(2):294–305. 26662117 10.1111/jth.13218

[31] Mahé I Chidiac J Bertoletti L Font C Trujillo-Santos J Peris M The Clinical Course of Venous Thromboembolism May Differ According to Cancer Site. Am J Med 2017;130(3):337–347. 27884650 10.1016/j.amjmed.2016.10.017

[32] Walker AJ West J Card TR Humes DJ Grainge MJ. Variation in the risk of venous thromboembolism in people with colorectal cancer: a population-based cohort study from England. J Thromb Haemost 2014;12(5):641–9. 24977288 10.1111/jth.12533 PMC4230392

